# Unexpected Placenta Accreta Spectrum During Cesarean Section: Intraoperative Diagnosis and Life-Saving Surgical Management

**DOI:** 10.7759/cureus.107473

**Published:** 2026-04-21

**Authors:** Anibri Mouna, Benabdeslam Rim, Fathi Khaliud, Bargach Samir

**Affiliations:** 1 Department of Obstetrics and Gynecology, Oncology and High-Risk Pregnancy, Souissi Maternity Hospital, Rabat, MAR

**Keywords:** abnormal placentation, cesarean section, hysterectomy, intraoperative diagnosis, maternal morbidity, obstetric hemorrhage, placenta accreta spectrum

## Abstract

Placenta accreta spectrum (PAS) is a major cause of obstetric hemorrhage and maternal morbidity. Despite advances in antenatal imaging, some cases remain undiagnosed until delivery. We report a case of intraoperative diagnosis of placenta accreta in a 32-year-old woman with a history of two prior cesarean sections. Following delivery of a healthy neonate, the placenta failed to separate and was found to be abnormally adherent to the uterine wall. In the setting of ongoing bleeding and absence of a cleavage plane, a subtotal hysterectomy was performed. The patient had a favorable postoperative outcome. This case underscores the importance of anticipating PAS in high-risk patients and highlights the need for rapid intraoperative decision-making when the diagnosis is unexpected.

## Introduction

Placenta accreta spectrum (PAS) refers to a group of conditions characterized by abnormal trophoblastic invasion into the myometrium due to defective decidualization [1]. It encompasses placenta accreta, increta, and percreta, depending on the depth of invasion [2].

The incidence of PAS has increased significantly over recent decades, largely due to the rising rate of cesarean deliveries [1,3]. This condition is associated with severe maternal morbidity, including massive hemorrhage, transfusion requirement, and peripartum hysterectomy [3,4].

Although antenatal diagnosis using ultrasound and magnetic resonance imaging has improved detection rates, PAS may still be diagnosed unexpectedly at the time of delivery, particularly in settings with limited prenatal care [5,6]. These situations pose significant intraoperative challenges and require rapid clinical decision-making.

## Case presentation

A 32-year-old woman, gravida 3 para 2, with a history of two previous cesarean sections, was admitted for an elective cesarean delivery at 38 weeks of gestation. Antenatal follow-up was irregular, and no imaging suggestive of abnormal placentation had been documented.

A cesarean section was performed, requiring a vertical uterine incision in the upper segment due to intraoperative findings. A healthy neonate was delivered without complications. However, during the third stage of labor, the placenta failed to separate spontaneously. Gentle traction on the umbilical cord was unsuccessful and resulted in suspicion of abnormal placental adherence.

**Figure 1:**
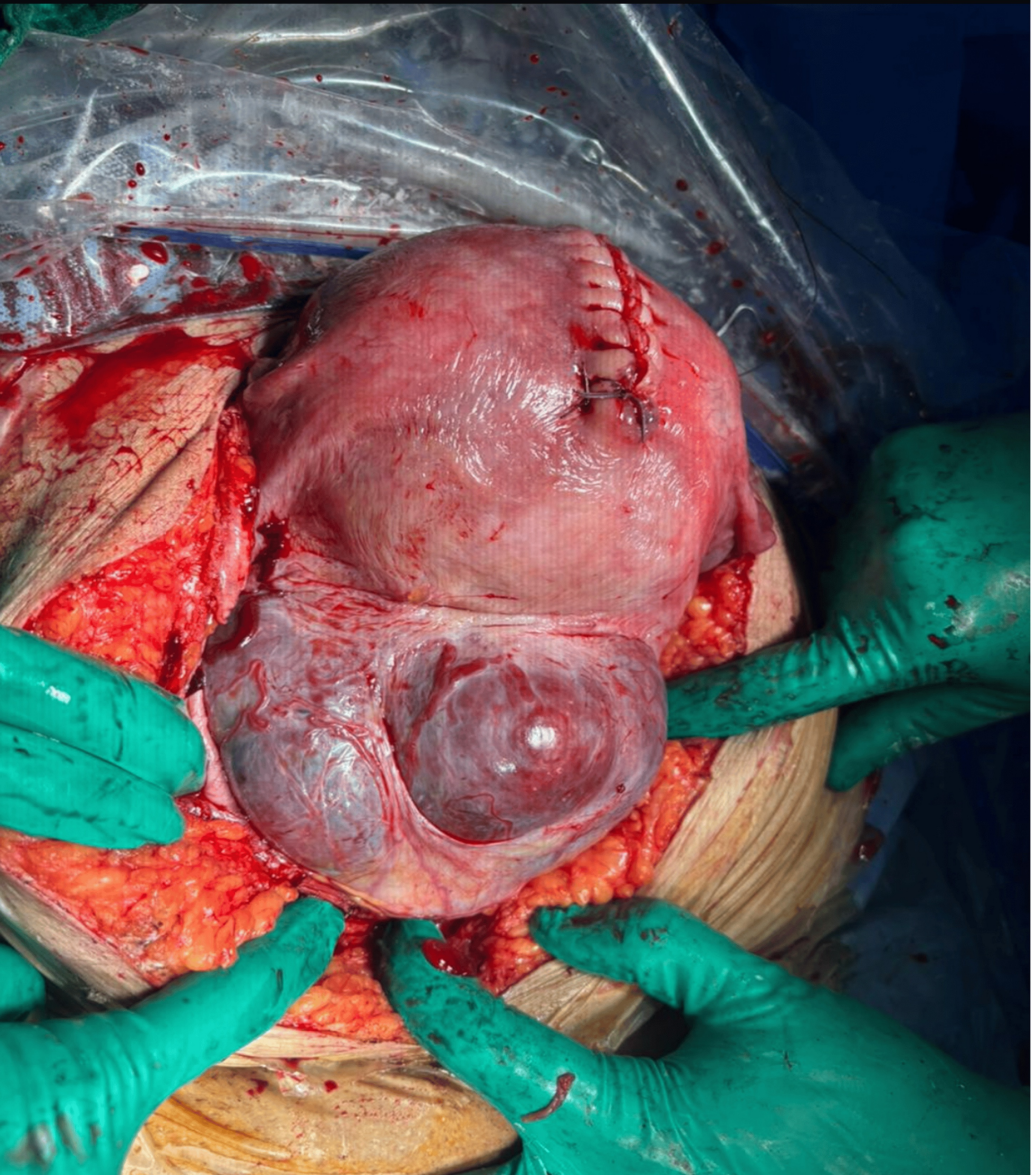
Intraoperative view showing abnormal placental adherence to the uterine wall with the absence of a cleavage plane, consistent with the placenta accreta spectrum.

On direct inspection, the uterus appeared enlarged and globular, with a visibly adherent placental mass and prominent vascularization. No clear cleavage plane could be identified between the placenta and the myometrium (Figure 1). At this point, further attempts at placental removal were considered unsafe. The intraoperative image (Figure 1) clearly demonstrates abnormal placental adherence with the absence of a cleavage plane and increased vascularity, supporting the diagnosis of PAS.

Given the intraoperative findings and hemodynamic concern, a decision was made to proceed with a subtotal hysterectomy as a life-saving measure due to ongoing hemorrhage, absence of a cleavage plane, and the need to reduce operative time in an emergency setting. The procedure was completed without major intraoperative complications.

Estimated blood loss was approximately 1500 mL. The patient received three units of packed red blood cells. Postoperative recovery was uneventful, and the patient was discharged on postoperative day five in stable condition.

## Discussion

This case illustrates a typical presentation of PAS diagnosed intraoperatively. A history of previous cesarean sections remains the most important risk factor, significantly increasing the likelihood of abnormal placentation [1,3,7].

Antenatal diagnosis is essential to optimize outcomes. Ultrasound findings suggestive of PAS include placental lacunae, loss of the retroplacental clear zone, and abnormal vascularization [2,5]. Magnetic resonance imaging can be useful in selected cases [6].

However, in daily clinical practice, particularly in settings with limited resources or inconsistent prenatal follow-up, such findings may be missed. The absence of a cleavage plane during placental separation is a key intraoperative sign. Attempting forcible placental removal can result in life-threatening hemorrhage and should be avoided [3,4].

Current guidelines recommend planned cesarean hysterectomy for confirmed PAS cases, ideally performed in a multidisciplinary setting [8]. When PAS is encountered unexpectedly, management depends on intraoperative findings and surgical expertise. Conservative approaches have been proposed in selected cases, but they remain associated with risks such as delayed hemorrhage and infection [9]. Optimal surgical strategies and timing remain key determinants of outcome [10].

Multidisciplinary management has been shown to improve maternal outcomes in complex PAS cases [11]. The pathophysiological mechanisms underlying abnormal placental invasion have been extensively described, providing further insight into PAS development [12].

Although total hysterectomy is generally recommended in cases of PAS, particularly when the placenta is low-lying, subtotal hysterectomy may be performed in emergency settings to reduce operative time and control hemorrhage.

## Conclusions

PAS is a life-threatening obstetric condition that may be diagnosed unexpectedly during a cesarean section. This case highlights the importance of maintaining a high index of suspicion in patients with prior uterine surgery. Rapid intraoperative recognition and timely hysterectomy remain key to preventing severe maternal morbidity and mortality.
